# Better Brain Health through Equity: Addressing Health and Economic Disparities in Dementia

**DOI:** 10.1093/geroni/igab046.3567

**Published:** 2021-12-17

**Authors:** Rajiv Ahuja, Cara Levy

**Affiliations:** Milken Institute, Washington, District of Columbia, United States

## Abstract

Dementia disproportionately impacts the health and financial security of women and certain minority groups. Long-standing inequities create distrust of the medical system, fewer treatment options, and reduced access to care. Research predicts that from 2020 to 2060, the number of African Americans and Latinx living with dementia will grow by nearly 200 percent and 440 percent, respectively, while prevalence among non-Hispanic Whites will increase by 69 percent. As the prevalence of dementia rises, so will the costs associated with dementia care. African Americans bear 1/3 of the costs associated with dementia. And the costs for Latinx living with Alzheimer’s disease are expected to exceed $100 billion by 2060. To mitigate these growing health and economic concerns, efforts to improve dementia care must put equity front and center. This presentation highlights five actionable recommendations to build health equity by reducing disparities in dementia prevention, detection, diagnosis, and care. These recommendations center around two overarching themes: (1) Strengthening the infrastructure among health-care, long-term care, and community-based organizations to achieve greater health equity for people living with dementia and their caregivers and (2) Expanding dementia-friendly networks and workplaces in racially and ethnically diverse communities. The recommendations discussed in this presentation will offer guidance for policymakers, health services researchers, businesses, health systems, and communities to reduce the inequitable impact of dementia on African Americans and Latinx, which is even more vital amid demographic trends showing a population growing older and more racially and ethnically diverse.

